# Effect of Injection Speed on Oocyte Deformation in ICSI

**DOI:** 10.3390/mi10040226

**Published:** 2019-03-29

**Authors:** Amir M. Hajiyavand, Mozafar Saadat, Alessandro Abena, Ferhat Sadak, Xiaochen Sun

**Affiliations:** Department of Mechanical Engineering, School of Engineering, University of Birmingham, Birmingham B15 2TT, UK; m.saadat@bham.ac.uk (M.S.); A.Abena@bham.ac.uk (A.A.); FXS587@student.bham.ac.uk (F.S.); XXS637@student.bham.ac.uk (X.S.)

**Keywords:** Intracytoplasmic Sperm Injection (ICSI), injection speed, injection force, cell deformation, Finite Element Method (FEM), vibrations

## Abstract

Oocyte deformation during injection is a major cause of potential cell damage which can lead to failure in the Intracytoplasmic Sperm Injection (ICSI) operation used as an infertility treatment. Injection speed plays an important role in the deformation creation. In this paper the effect of different speeds on deformation of zebrafish embryos is studied using a specially designed experimental set-up. An analytical model is developed in order to link injection force, deformation, and injection speed. A finite element (FE) model is also developed to analyse the effect of injection speed, allowing the production of additional information that is difficult to obtain experimentally, e.g., deformation and stress fields on the oocyte. The numerical model is validated against experimental results. Experimental results indicate that by increasing the injection speed, the deformation decreases. However, higher speeds cause higher levels of injection force and force fluctuation, leading to a higher vibration during injection. For this reason, an optimum injection speed range is determined. Finally, the FE model was validated against experimental results. The FE model is able to predict the force-deformation variation during injection for different speeds. This proves to be useful for future studies investigating different injection conditions.

## 1. Introduction

Biological cell injection is a laborious process, which introduces a foreign material into a biological cell with various applications such as Preimplantation Genetic Diagnosis (PGD), Intracytoplasmic Sperm Injection (ICSI), and embryo biopsy. In ICSI, the current oocyte injection procedures are suffering from low success rates. The conventional method of oocyte injection is operated by a human who relies on visual feedback from an optic device. The conventional method is performed manually using two manual joysticks. In this method, the tip of the injection micropipette is moved towards the oocyte to the point where it touches the zona pellucida and then the oolemma membranes. Afterwards, it moves rapidly until it pierces the membrane and delivers foreign material into the desired location in the oocyte [1,2]. This method is prone to errors as it relies on the skills of an operator who is guided by optical devices. During oocyte injection, there are some factors which are not possible to control manually using joysticks and which have a high impact on the success rate of the procedures. During the injection procedure the injection micropipette creates excessive deformation of the oocyte. Excessive deformation may induce emission of the cytoplasm into the perivitelline space after injection [3]. This causes an increase in the internal pressure of the oocyte and therefore leads to oocyte degeneration. Other causes of the low success rate are excessive force, which causes additional deformation, and hand tremor. Automating the entire process has been the main focus in the literature [4,5] in order to eliminate human involvement and therefore deformation due to excessive cellular force.

Automating and mechanising the whole process aside including haptic feedback to the operator in the ICSI operation has been reported in [6,7,8], where it is possible to observe that an haptic information allows to the operator to minimize physical damage to the cellular structure and increase the success rate of the embryo development procedure post injection. Since biological cells are highly deformable and irregular in conformation, they could be damaged seriously during non-controlled injection procedure [9]. Various force sensing techniques were proposed by researchers to improve cell survivability rates in the microinjection process [10,11]. Cellular force measurement is also essential for evaluating the stiffness of the biological cell membrane, which exhibits viscoelastic behaviour during the injection process, and a 3D mathematical model can be derived [12,13]. Therefore, having force feedback is a prior concern in micro injection tasks so that the operator can achieve better control over the micro injection and manipulation processes [14,15,16].

Although a combination of vision and force feedback provided to operator induce a higher success rate in oocyte injection tasks compared to visual feedback alone [15], regulating the penetration force at the time of injection is equally important to ulteriorly increase the embryo survival rate [17]. For this reason, Precise real-time force sensing is a requirement for reliable automatic cell injection processes [18]. However, the actual role of the indentation force on oocyte deformation and how this affects the success rate of the injection task remains deficient.

Despite the great developments above, numerous factors playing considerable roles in the process have not been entirely investigated while improving the microinjection task. Sufficient penetration force, penetration speed, and an accurate injection point are the key factors to achieve successful penetration in the microinjection process, which requires sensitive control methods [19]. The roles of microinjection speed and force feedback on cell deformation in cell penetration tasks are well recognized [20]. However, it has not been comprehensively studied.

Following the role of contact force in micro injection and how it affects the outcome of the system, analysing the effect of injection speed in microinjection on oocyte deformation is a must to reduce the deformation of a oocyte and improve embryo survivability rate. Since the injection micropipette is in contact with a cell at different indentation speeds, the contact force and deformation rate are also changing. There is a complex dynamical relation among contact force, injection speed, and oocyte deformation. Although speed analysis in microinjection is a promising way to obtain better outcomes in the context of getting less deformation on cells in comparison to constant injection speed [20], force and speed analysis on deformation creation in oocyte injection tasks remain a gap in the literature.

The present study aims to analyse the effect of the injection speed on cell deformation, injection force, and force fluctuation by means of in-house experiments. In addition, two predictive models are presented and validated against experimental findings: an analytical model and a numerical model. The former employs a dynamic model to obtain a relation between injection force, cell deformation, and velocity. The latter implements the finite element method (FEM) to simulate the injection process, obtaining also information that could be difficult to obtain experimentally (e.g., stress distribution and three-dimensional deformation field on the cell). After validation, the FEM model can then be used for further research on the biological cell injections avoiding to actually carry out the experiment.

## 2. Materials and Methods

In this section, details on experimental design and on development of predictive models are provided. In particular, two different models are presented: an analytical model and a FE model.

The numerical model is developed to provide the predictive information for force and deformation during various injection speed ranges. To validate the model in a wider range, it was essential to measure the highest and lowest possible injection speeds, which were 0.05 mm/s and 1 mm/s, respectively [20]. Controlling the injection operation in the speeds over 0.6 mm/s has proved to be challenging due to the cell size and high fluctuations and vibrations occurring during injection in the current set up [21]. This is deemed to cause potential damage to the cell. Consequently, it has been decided to measure the deformation, force, and fluctuations within a reasonable range of 0.05 to 0.6 mm/s under controlled operational conditions. At higher speeds a sudden increase in fluctuation (up to 55%) is experienced, preventing the injection to be conducted under stable conditions. Considering this, and in order to validate the numerical model, only one velocity has been examined above 0.6 mm/s, which is 1 mm/s.

### 2.1. Experimental Design and Test Rig

**Figure 1:**
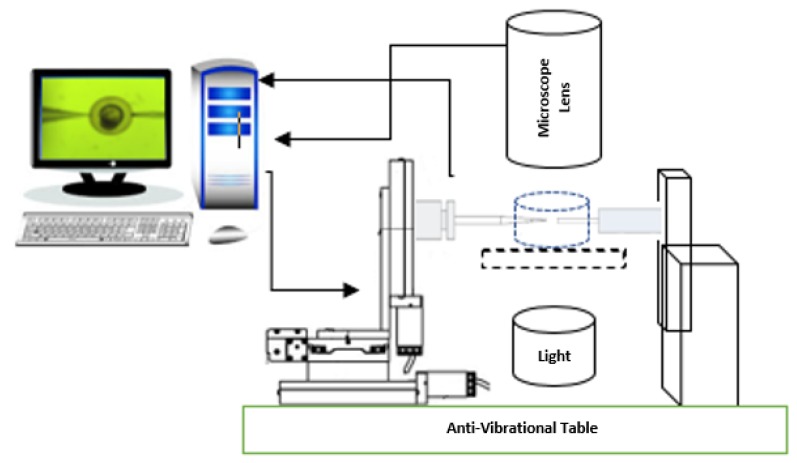
Schematic view of the experimental set up.

The experimental setup comprises of a precision injection system integrated with an accurate and sensitive loadcell to measure the indenting force of a zebrafish embryo. A data acquisition device is employed to convert the analogue outputs from the load cell to digital. The obtained data are analysed using computer software and raw data has been defined as force unit. A SS2 Sherborne Ultra Low Force Load Cell with capacity of 0.5886 N and resolution of 0.1 mN is utilised for measuring the membrane reaction force during the injection. Figure 1 shows the schematic of the experimental set up.

**Figure 2:**
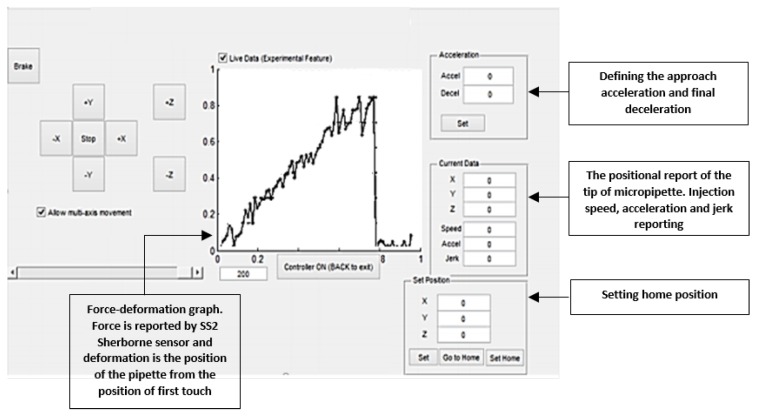
Graphical user interface for the injection system.

Figure 2 shows the graphical user interface (GUI) for controlling the injection system. This GUI can control the coarse and fine movement of the stage, enabling us to control the injection speed. The recorded force is also shown as graph on this window during the injection procedure. The software allows adjusting acceleration, deceleration, and injection speed.

Microinjection of zebrafish is commonly employed in research for validating the early developmental processes as it reveals viscoelastic properties during the injection procedure. Zebrafish embryo is one of the usual animal models which is well-stablished in microinjection technique in science and biomedical technologies [5,12,22,23,24]. In biological research the zebrafish embryo is a reliable model for the mechanical tests and response behaviour analysis. Additionally, it is a dominant model to realize the genes’ role during the development stage. The other advantages of the zebrafish embryo are transparency, high productivity, and being of a decent size for mechanical experiments. In this research, zebrafish embryos were collected freshly with the protocol mentioned in guidance for the laboratory use of zebrafish. The embryo samples were transferred to the laboratory on the days of the experiments [25].

**Figure 3:**
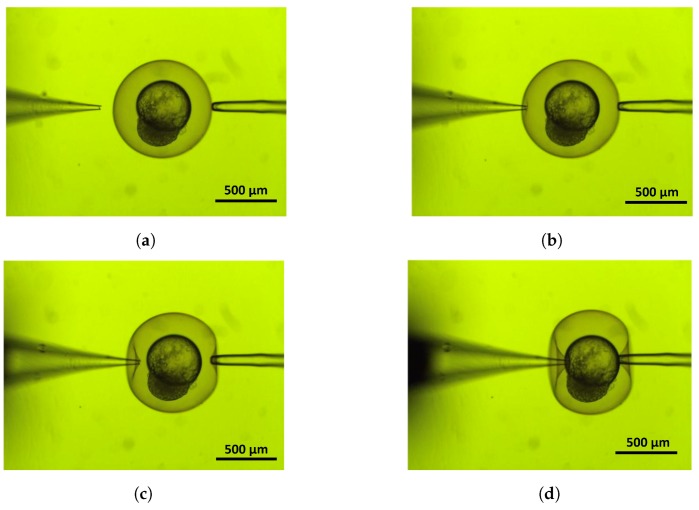
Cell deformation for different stages of zebrafish injection: (**a**) positioning, (**b**,**c**) deforming, (**d**) puncturing.

During the experiment, a sample dish is placed within the focal zone of microscope. Then the oocyte is restrained and the injection pipette is set toward the centre of the embryo within a certain distance from the chorion. This distance allows the injector to achieve the desired speed before the tip of the pipette touches the chorion. The injection micropipette stops the motion once the breakage of membrane reports a sudden drop of the recoded indentation force. The total deformation and position of the cell were reported to the controller using visual feedback [26,27]. Figure 3 indicates different stages of the injection for a zebrafish embryo. The size for the zebrafish embryo is the average size of 600–1200 μm and the pipette radius is 30 μm [5,23].

Five different injection speeds were investigated: 0.05 mm/s, 0.1 mm/s, 0.2 mm/s, 0.4 mm/s, and 0.6 mm/s. The injection was repeated ten times for each speed on different embryos. Deformation and injection force were measured for each injection, and the average value for each speed was then calculated.

### 2.2. Analytical Model

The analytical model of injection procedure employs a dynamic model to determine the relation between injection speed, deformation, and injection force.

In the model development the following assumptions have been considered:

- the cell is considered as having a fully spherical shape;
- a uniform hydrostatic pressure is applied on the cell membrane;
- the thickness of the cell membrane is considered uniform before the injection;
- the cytoplasm inside is defined as incompressible and the oocyte volume has been kept uniform;
- the elasticity of the membrane changes linearly.

Deformation of the oocyte membrane depends on the dynamics of injection, e.g., injection speed, and on the unique characteristics of the individual oocyte at the time of injection, e.g., morphological shape of the oocyte and maturation level.

**Figure 4:**
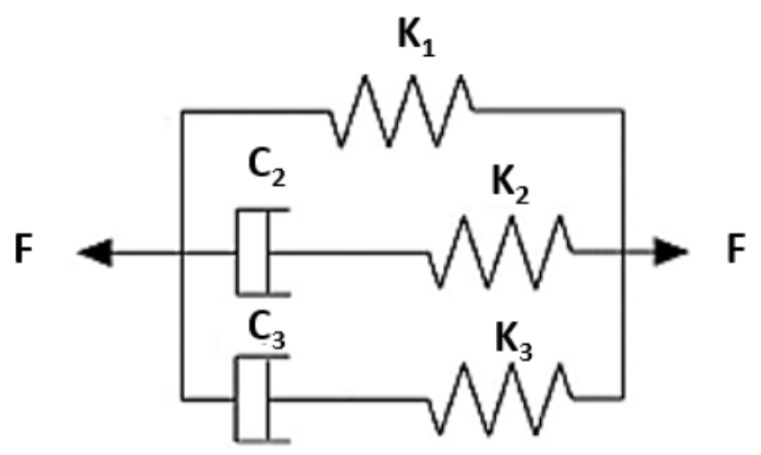
Schematic of Maxwell–Wiechert Model.

The Maxwell–Wiechert model is employed to demonstrate the oocyte response behaviour. The adopted model contains two standard linear forms of Maxwell in parallel to one spring, as shown in Figure 4. The Hookean springs and Newtonian dashpots represent the elastic and viscoelastic behaviours of the oocyte, respectively.

The spring and dashpot equations are written as following:

(1) F=KX

(2) $$F=CV=C\frac{dX}{dt}$$

where coefficients *K* and *C* represent spring and damper constants, respectively.

During the insertion of the injection micropipette, all arms are compressed by a $X_{t}$ amount, which represents the value of the deformation. On an arm of Maxwell, the deformation $X_{t}$ represents the sum of spring $X_{s}$ and damper $X_{d}$ deformations. The force acting on each arm of Maxwell is calculated as following:

(3) $$F\left(t\right)=KX_{t}\exp\left(-\frac{Kt}{C}\right)$$

Since the force acting on spring and dashpot is the same, then:

(4) $$KX_{s}=C\frac{dX_{d}}{dt}$$

Hence, Equation (3) can be rewritten as following:

(5) $$F\left(t\right)=CV\exp\left(-\frac{Kt}{C}\right)$$

Finally, the resultant force for Maxwell–Wiechert model results:

(6) $$F\left(t\right)=K_{1}X_{t}+C_{2}V\exp\left(-\frac{K_{2}t}{C_{2}}\right)+C_{3}V\exp\left(-\frac{K_{3}t}{C_{3}}\right)$$

Equation (6) represents the relation between injection speed, deformation and injection force, where coefficients $K_{1}$, $K_{2}$, $K_{3}$, $C_{2}$, and $C_{3}$ are calibrated based on results obtained from in-house experiments.

**Figure 5:**
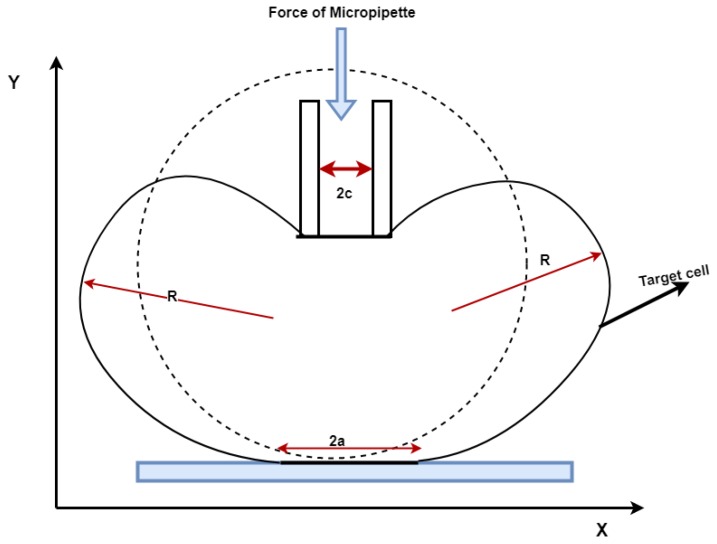
Point load model of injection [28].

Finally, the elastic modulus *E* of the chorion of zebrafish can be calculated using Equation (7) [28], which is based on the point load model of injection as shown in Figure 5:

(7) $$F=\frac{2\pi Eh\omega_{d}^{3}}{a^{2}\left(1-\theta \right)}\left[\frac{3-4\zeta^{2}+\zeta^{4}+2\ln\zeta^{2}}{\left(1-\zeta^{2}\right){\left(1-\zeta^{2}+\ln\zeta^{2}\right)}^{3}}\right]$$

where ζ=c/a, with *a* and *c* geometric parameters obtained by means of image analysis, *F* is the injection force, *h* is the membrane thickness (chorion thickness), ω is the deformation depth, and θ is the Poisson’s ration.

### 2.3. Finite Element Model

A finite element model was developed to analyse the effect of different injection speeds on oocyte deformation and to predict the injection force.

A three-dimensional finite element model was developed using Abaqus 6.13. This model employed explicit dynamical analysis to simulate zebrafish embryo indentation. Three different indentation speeds were considered: 0.1 mm/s, 0.6 mm/s, and 1 mm/s. These are considered as low, medium, and high injection speeds, respectively. In particular, the lowest and the highest injection speed were used to calibrate the model, while the medium speed for validation purpose. A total of 4287 shell elements (S4R elements) were used to simulate the embryo. Injection micropipette and the holding pipette were simulated as rigid bodies, due to the fact that their Young’s modulus is very high when compared to that of the oocyte. This simplification allows to reduce the computational cost of the simulation and it has been used in several fields, from ballistic impact problems [29] to machining processes [30,31]. A finer mesh was used near the contact points of the oocyte with the injection micropipette and the holding pipette, with a decreasing mesh density moving away from these areas. The thickness associated with the shell elements was set to 3 μm. The radius of the oocyte and injection micropipette were 400 μm and 30 μm, respectively. A pressure of 2.5 KPa was then applied to the contact area between the holding micropipette and the oocyte to simulate the suction pressure necessary to maintain the oocyte in place during the injection.

The general contact algorithm was utilised to simulate the contact among different parts of the model; and the Coulomb friction model was used with a friction coefficient of 0.3. The friction allows maintaining the oocyte in position, preventing it from slipping due to the compression force applied by the injection micropipette.

**Figure 6:**
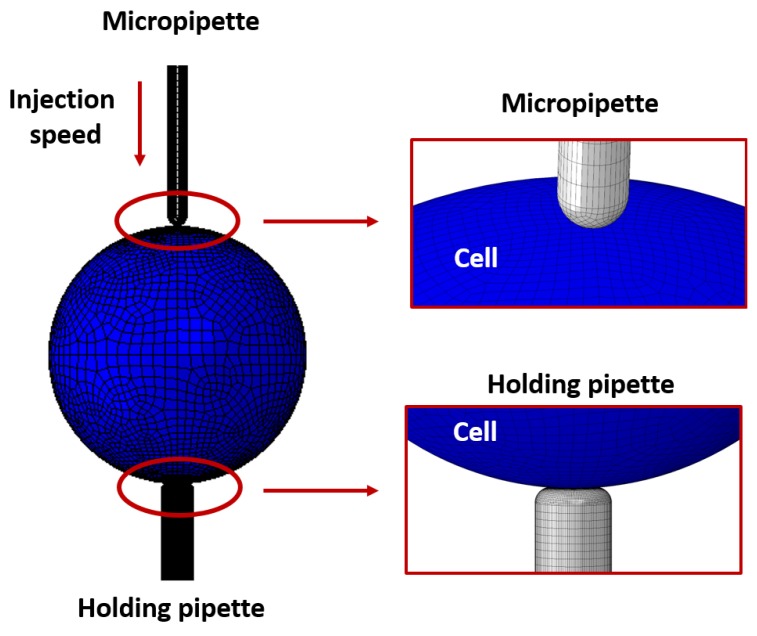
Schematic of finite element (FE) model for Intracytoplasmic Sperm Injection (ICSI).

The oocyte was simulated as linear elastic. A Poisson ratio of 0.3 was used in the simulation. The Young’s modulus was implemented as a function of the injection speed, and calibrated based on experimental results for injection speeds of 0.1 mm/s and 1 mm/s. The necessity of a calibration for the young modulus of the oocyte can be explained considering the high computational cost associated with micro-mechanical models. Since the parameter of interest in the present study is the deformation of the external membrane of the oocyte, in order to drastically reduce the computation time of the analysis, the oocyte was considered as an empty shell positioned between the injection micropipette and the holding pipette. Therefore, the Young modulus of the oocyte’s membrane needs to be calibrated in order to make the oocyte deforming as observed in the experiment and to obtain values of the injection force close to those measured in experiments, where the oocyte is not an empty shell. The developed model is shown in Figure 6.

## 3. Results and Discussion

This section presents and discusses the experimental results and the calibrated analytical and FE models for various injection speeds.

### 3.1. Experimental Results

**Figure 7:**
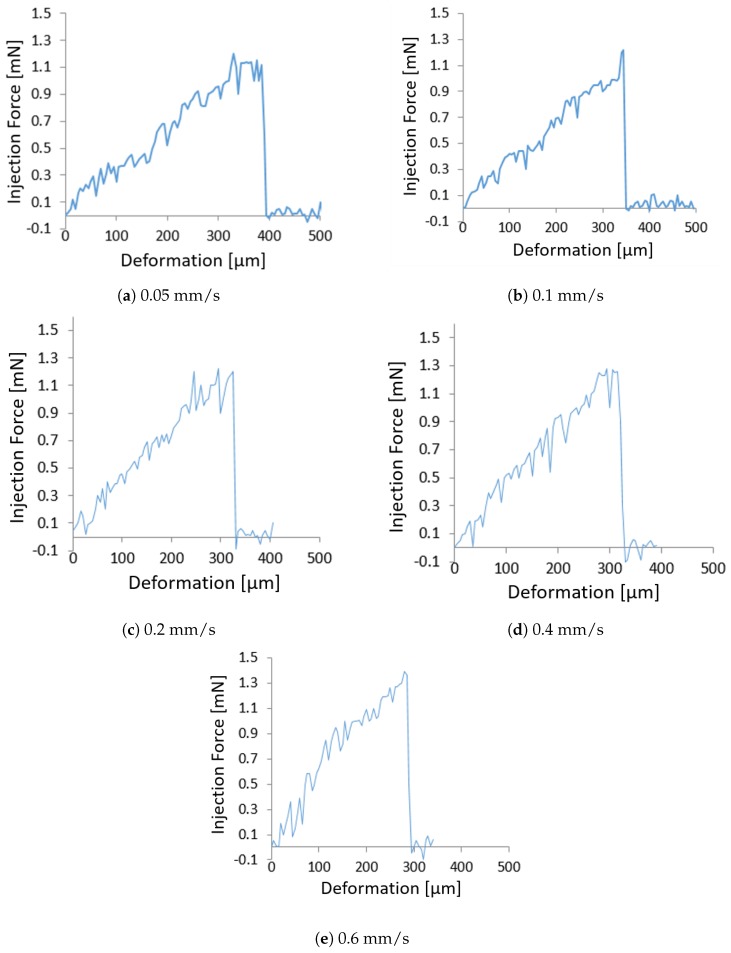
Experimental results in terms of force-deformation for various injection speeds.

The recorded force is presented versus deformation in Figure 7 for all different injection speeds investigated. Each graph in Figure 7 represents the average of the graphs obtained from ten experiments at a constant speed. It is possible to observe that the rate of increase in force grows with the injection speed increase, causing less deformation and high injection force.

Experimental results are reported in Table 1 in terms of deformations at penetration (injection micropipette pierces the oocyte) and indentation forces for all different velocities for a zebrafish embryo.

From Figure 7 and Table 1, it is possible to observe that an increase of injection speed causes an increase of the indentation force and a decrease of deformation exerted on the oocyte at puncture. In fact, deformation decreases by approximately ∼34% and force increases by ∼16% when injection speed increases from 0.05 mm/s to 0.6 mm/s.

**Figure 8:**
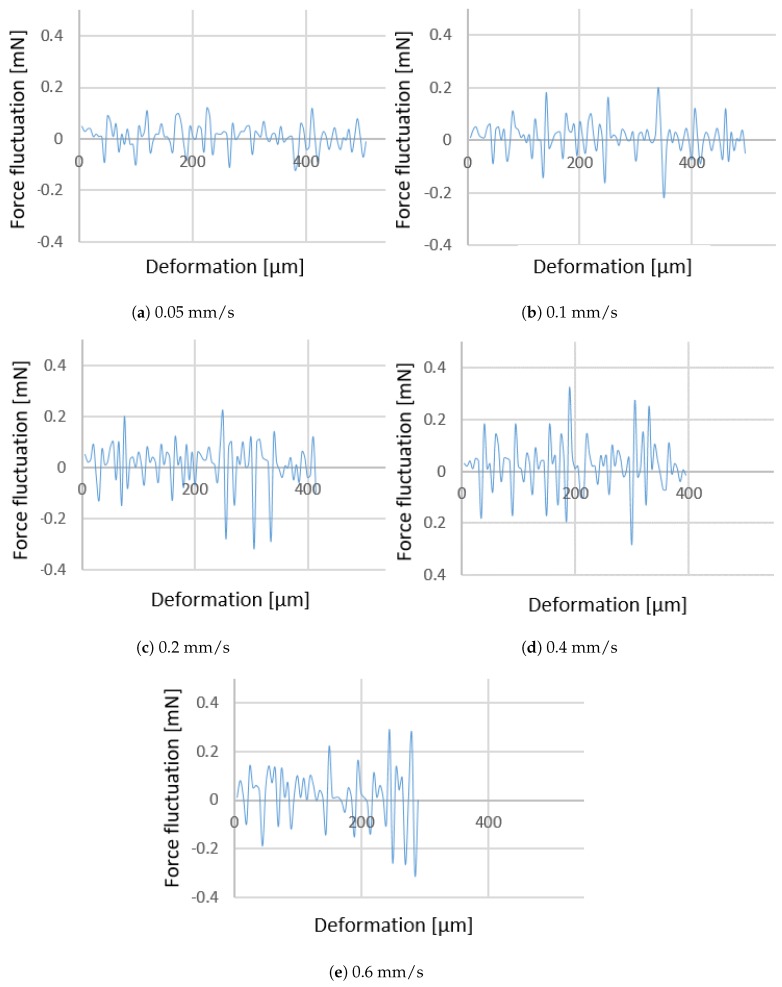
Force fluctuation obtained for varius injection speeds.

Increasing the injection speed, the force fluctuation increases as shown in Figure 8. The fluctuation of the force is representative of the amount of vibrations during injection. In particular, the lateral and axial vibrations during injection cause the force fluctuation. Controlling the injection operation in the speeds over 0.6 mm/s has proved to be experimentally challenging due to the cell size and high fluctuations and vibrations occurring during injection. This is deemed to cause potential damage to the cell. At higher speeds a sudden increase in fluctuation (up to 55%) is experienced, preventing the injection to be conducted under stable conditions. Considering this, only one velocity has been examined above 0.6 mm/s, which is 1 mm/s in order to calibrate the numerical model.

**Figure 9:**
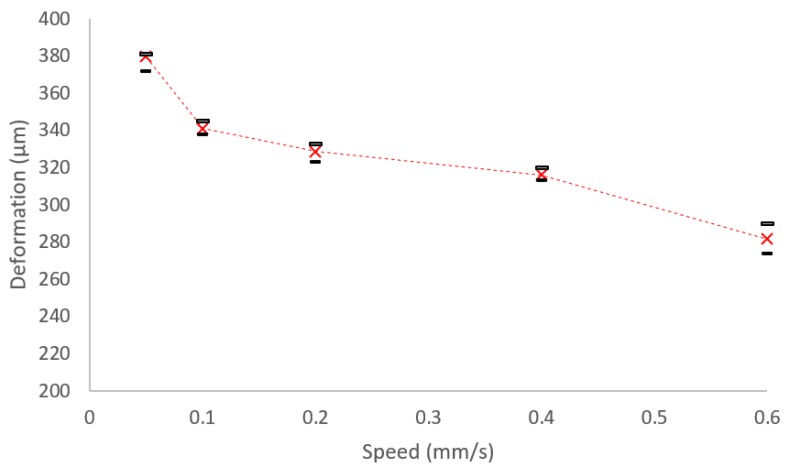
Deformation of Zebrafish embryo using different constant injection speeds.

The experimental results of the injection speed versus deformation at puncture are presented in Figure 9. As previously observed, the deformation reduces with an increase in the injection speed. However, it is possible to observe that the rate of reduction when increasing the injection speed is not constant.

**Figure 10:**
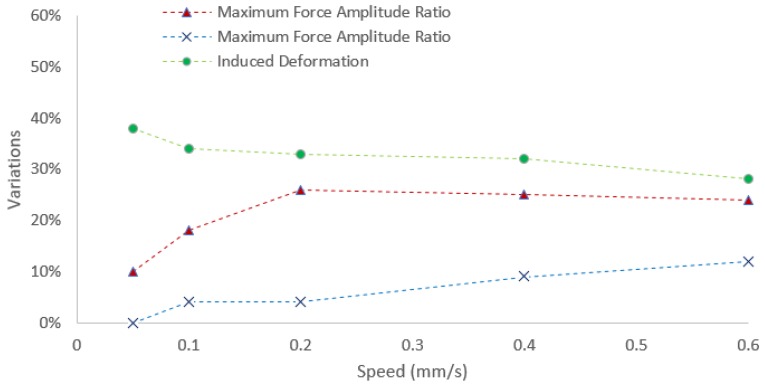
Influence of injection speed on oocyte deformation, injection force, and force fluctuation.

Figure 10 compares the variations in Maximum force amplitude ratio, penetration force, and induced deformation on the membrane among various injection speeds. Maximum force amplitude ratio (MFAR) is a ratio of the maximum amplitude recorded during the injection to the maximum force (the indentation force) reported in the same set, which is represented in percentage. This variable indicates that maximum fluctuation happens during the injection which indicates the total force changing based on vibration. The penetration force graph indicates the ratio of the maximum force (penetration force) compare to the penetration force reported for speed of 0.05 mm/s. However, the induced deformation graph shows the proportion of deformation to the total diameter ratio of the zebrafish embryo. This graph demonstrates the total behaviour of three important injection factors due to speed variations all in percentage. As it is demonstrated in the table reported in Figure 10, the maximum force amplitude ratio reported is almost 25% for the first five sets which have injection speeds less than 0.6 mm/s. Although the MFAR and indentation force have been slightly increased during the first five sets, deformation decreased by 10%, which is almost 100 μm for set 5.

### 3.2. Analytical Model Results

The analytical model is based on Equation (6), which links injection force, deformation, and velocity. It has been applied to the final stage of the injection to determine the force at which the puncture takes place. Hence, the five unknown coefficients were determined through a model calibration based on the experimental results. Values of calibrated coefficients are reported in Table 2.

Based on values of coefficients reported in Table 2, Equation (6) can be written as following:

(8) $$F\left(t\right)=\left[0.3X_{t}+0.125V\exp\left(-\frac{0.356t}{0.125}\right)+4.74V\exp\left(-\frac{0.668t}{4.74}\right)\right]\frac{1}{100}$$

where $X_{t}$ represents the deformation at which the injection micropipette breaks the membrane.

**Figure 11:**
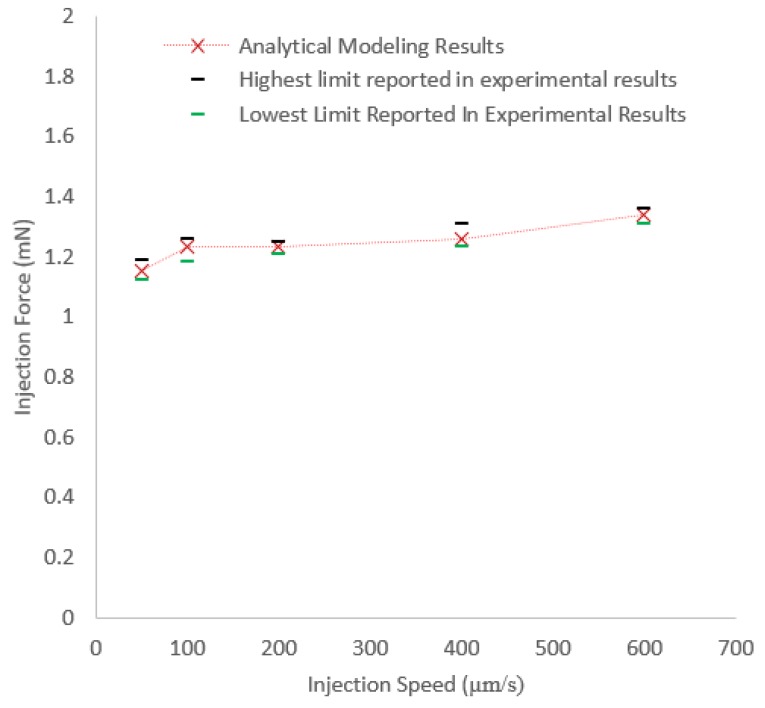
Comparison between experimental and analytical results.

Figure 11 shows the comparison of the results calculated from analytical modelling, using Equation (8), and the maximum and minimum injection force obtained from experiments. The results indicates the accuracy of the developed analytical model in predicting the injection force.

Finally, the Young’s modulus of the chorion has been calculated according to Equation (7) by means image analysis, considering a membrane thickness of 3 μm and a Poisson’s ratio of 0.5. The average value of the Young’s modulus over all the experiments carried out is 1.32 ± 0.23 MPa (Table 3), which is close to the value reported in the literature (1.51 ± 0.07 MPa) [32].

### 3.3. Finite element model results

A FE model has been proposed for modelling the injection process. The model was calibrated in terms of Young modulus using the experimental results for a low (0.1 mm/s) and a high (1 mm/s) injection speed, aiming to obtain an injection force and a oocyte deformation as close as possible to those measured and observed experimentally. Young modulus values obtained from calibration are reported in Table 4, where $E_{0.1}$ and $E_{1}$ represent the Young modulus for injection speed of 0.1 mm/s and 1 mm/s, respectively.

**Figure 12:**
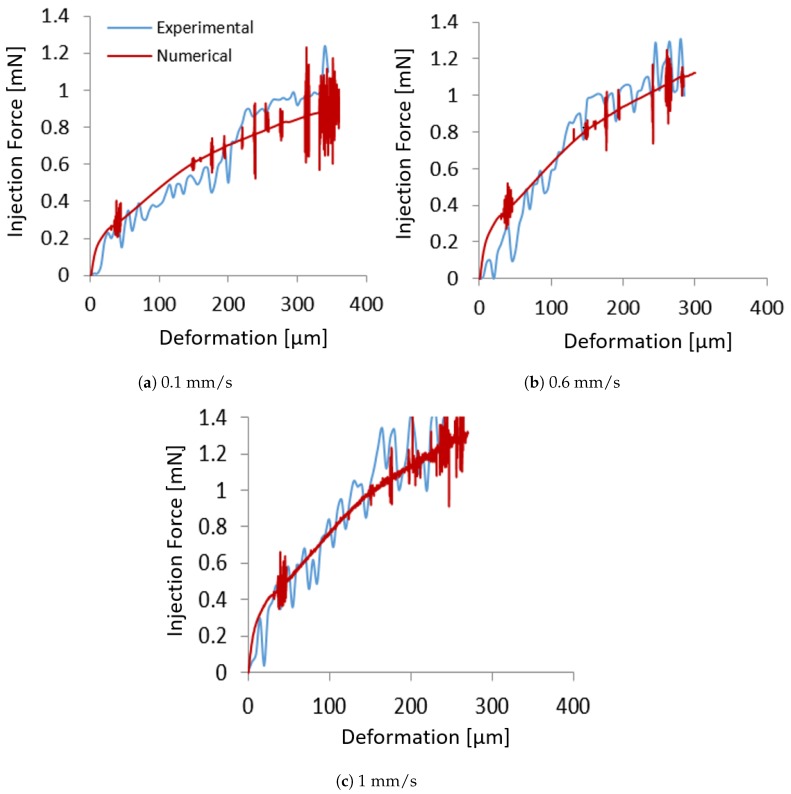
Comparison between experimental and numerical results in terms indentation force vs deformation for three various speeds: (**a**) 0.1 mm/s, (**b**) 0.6 mm/s, and (**c**) 1 mm/s.

Numerical results obtained for injection speed of 0.1 mm/s and 1 mm/s are compared with experimental findings in Figure 12a,c in terms of the force-deformation graphs. It is possible to observe as the numerical results are in good agreement with the experimental results, being able to accurately predict the force-deformation curve for both low and high injection speeds.

**Figure 13:**
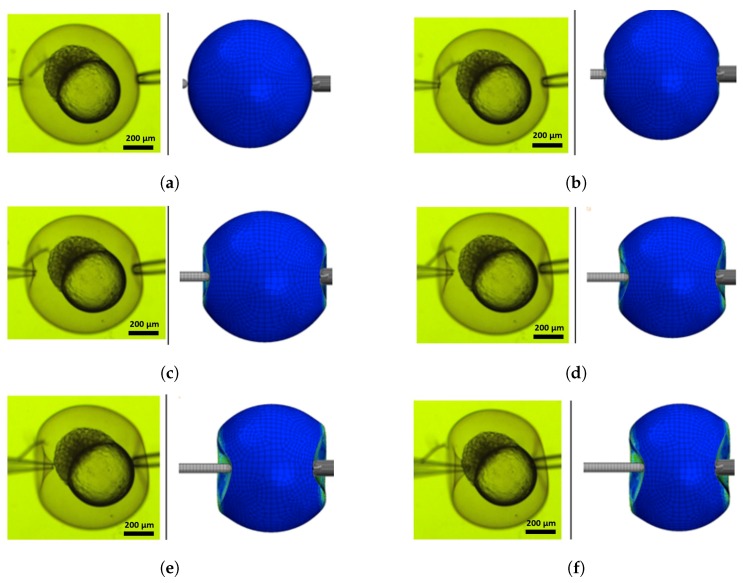
Comparison between experimental and numerical results for different stages of an injection process for injection speed of 0.1 mm/s: (**a**) positioning, (**b**–**e**) deforming, and (**f**) puncturing.

The different stages of an oocyte injection are shown in Figure 13, where a comparison between experimental and numerical results is reported for injection speed of 0.1 mm/s. Results show that the numerical model is able to predict the oocyte deformation during injection in the three-dimensional space. In particular, it is able to capture the deformation caused by the injection micropipette motion and also the one due to the holding pipette on the opposite side.

**Figure 14:**
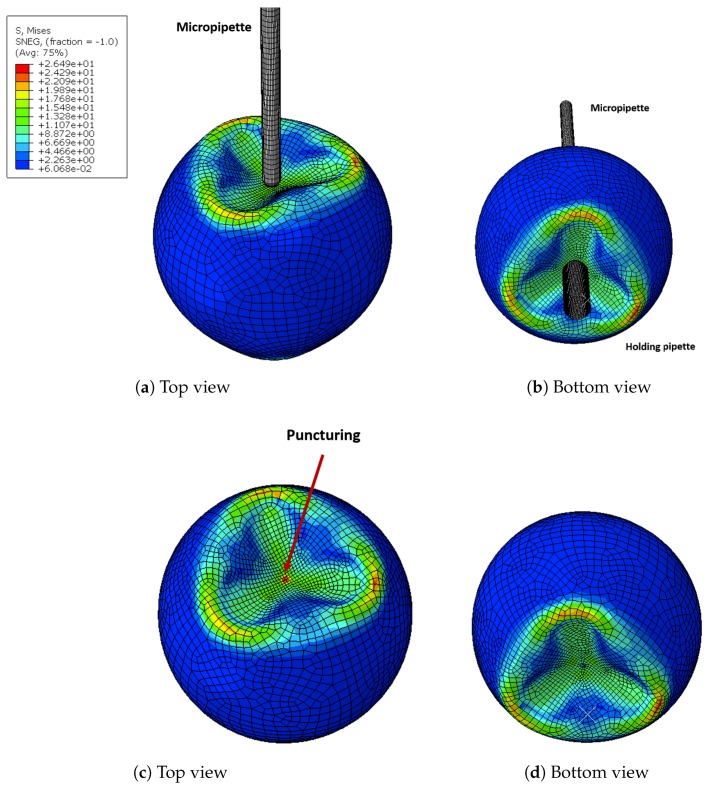
FE model results in terms of deformation field for injection speed of 0.1 mm/s.

The deformation the oocyte undergoes during injection is shown in Figure 14. The numerical model shows a non axial-symmetric deformation in the areas close to the injection micropipette and the holding micropipette. This deformation filed can be predicted only using a three-dimensional model. Von Mises stress is also shown in Figure 14. It is possible to observe that the highest stress value is located at the contact point with the injection micropipette, which causes the puncturing of the oocyte. Stresses due to the holding pipette are more contained due to its shape, which is flat on the top.

For any other injection velocity, a linear variation of the Young modulus was then assumed between the two velocities used for calibration. Hence, for a different injection speed *V* the Young modulus can be calculated by means of Equation (9).

(9) $$E_{V}=\left(\frac{V-0.1}{0.9}\right)200+300$$

In order to validate the numerical model, an injection speed of 0.6 mm/s was considered. Using Equation (9), the Young modulus was calculated to be ∼400 MPa. Results obtained in terms of force-deformation were compared with experimental findings and shown in Figure 12b. The numerical model is able to accurately predict the injection force and the deformation of the oocyte during injection, providing reliable results.

**Figure 15:**
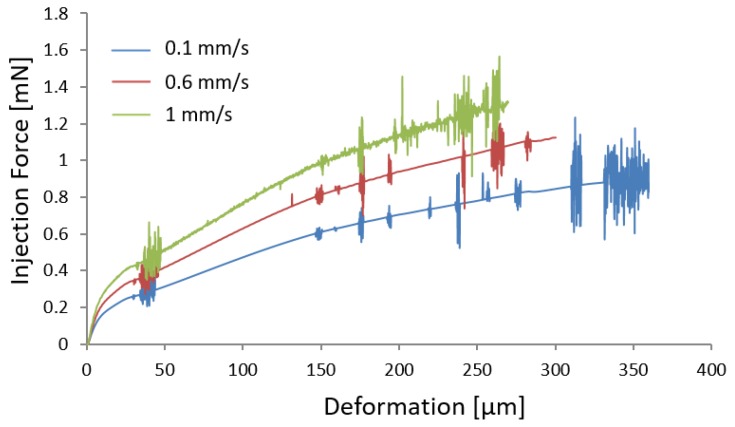
Comparison of the FE results for three injection speeds (0.1, 0.6, 1 mm/s).

Figure 15 indicates the force-deformation graphs obtained by the FE model for the three different injection speeds examined. Numerical results indicate that an increase of the injection speed causes a reduction of deformation at puncture, an increase of the injection force, and an increase of fluctuations. These findings are in agreement with what was observed experimentally. In fact, increasing the injection speed, the force increases as well (Table 1), leading also to a considerable fluctuation (Figure 8).

The developed model can then be used for investigating the injection process at different velocities, also providing information that cannot be obtained experimentally, e.g., stress distribution over the oocyte during injection.

Finally, dimensions and properties used in the model are summarised in Table 5.

## 4. Conclusions

In this article, the effect of the injection speed on oocyte deformation and injection force during Intracytoplasmic Sperm Injection of a zebrafish embryo has been studied experimentally. The range of injection speeds investigated was between 0.05 mm/s and 1 mm/s. The results show that a low injection speed is desirable to reduce the injection force, while a high speed has to be used for reducing oocyte deformation. In oder to obtain the optimum velocity, the force fluctuation represents a critical factor. In fact, the magnitude of force fluctuation has to be lower than 25% of the total force. This condition is satisfied only for injection speed less 0.6 mm/s. However, the optimum speed is a function of the particular system’s dynamic characteristics and, as such, in our experimental rig the optimum speed is determined to be within 0.4–0.6 mm/s.

An analytical model and a FE model have also been developed and validated against experimental results. The former was calibrated to be able to link injection speed, injection force, and deformation at the moment of puncture. The latter proved to be able to predict the force-deformation trend over the whole range of injection speeds investigated. In addition, it provided additional information on the stress and deformation fields over the oocyte during injection. In particular, a stress concentration was observed in the contact area between the injection micropipette and the oocyte, which causes oocyte breakage. The deformation field was found to be not axial-symmetric, suggesting to use a three-dimensional model when studying ICSI. The model can be further adapted for oocyte deformation analysis in ICSI.

## Figures and Tables

**Table 1:** Experimental results: injection speed, deformation, and indentation force.

Speed [mm/s])	Deformation [μm]	Indentation force [mN]
0.05	379.5	1.145
0.10	341.0	1.230
0.20	328.5	1.190
0.40	316.0	1.260
0.60	281.5	1.335

**Table 2:** Calibrated springs and dashpots constants for the adopted Maxwell–Wiechert model.

Coefficient	Value
$K_{1}$	0.3
$K_{2}$	0.356
$K_{3}$	0.668
$C_{2}$	0.125
$C_{3}$	4.74

**Table 3:** Comparison between experimental result and literature for the Young’s modulus.

Category	Stage	Young Modulus [MPa]	Standard Deviation [MPa]
**Literature**	Blastula	1.51	0.07
**In-house experiment**	Blastula	1.32	0.24

**Table 4:** Young modulus of the oocyte in the finite element (FE) model for injection speed of 0.1 mm/s and 1 mm/s.

Young modulus	Value [MPa]
$E_{0.1}$	300
$E_{1}$	500

**Table 5:** Dimensions and properties used in the numerical model.

Property	Value
Cell diameter	800 μm
Cell membrane thickness	3 μm
Young modulus $E_{0.1}$	300 MPa
Young modulus $E_{0.6}$	400 MPa
Young modulus $E_{1}$	500 MPa
Poisson ratio	0.3

## Abbreviations

The following abbreviations are used in this manuscript:

FEM	Finite Element Method
MFAR	Maximum Force Amplitude Ratio
ICSI	Intracytoplasmic Sperm Injection

PGD	Preimplantation Genetic Diagnosis
GUI	Graphic User Interface

## References

[1] Takeuchi S., Minoura H., Shibahara T., Shen X., Futamura N. (2001). Comparison of piezo-assisted micromanipulation with conventional micromanipulation for intracytoplasmic sperm injection into human oocytes. Gynecol. Obstet. Investig..

[2] Collas P., Barnes F. (1994). Nuclear transplantation by microinjection of inner cell mass and granulosa cell nuclei. Mol. Reprod. Dev..

[3] Yanagida K., Katayose H., Yazawa H., Kimura Y., Konnai K., Sato A. (1999). The usefulness of a piezo-micromanipulator in intracytoplasmic sperm injection in humans. Hum. Reprod..

[4] Lu Z., Zhang X., Leung C., Esfandiari N., Casper R., Sun Y. (2011). Robotic ICSI (Intracytoplasmic Sperm Injection). IEEE Trans. Biomed. Eng..

[5] Wang W., Liu X., Gelinas D., Ciruna B., Sun Y. (2007). A fully automated robotic system for microinjection of zebrafish embryos. PLoS ONE.

[6] Ghanbari A., Chen X., Wang W., Horan B., Abdi H., Nahavadi S. Haptic microrobotic intracellular injection assistance using virtual fixtures. Proceedings of the 11th International Conference Control Automation Robotics & Vision, ICARCV.

[7] Sieber A., Valdastri P., Houston K., Eder C., Tonet O., Menciassi A., Dario P. (2008). A novel haptic platform for real time bilateral biomanipulation with a MEMS sensor for triaxial force feedback. Sens. Actuators A Phys..

[8] Ammi A., Ferreira A. (2006). Biological cell injection visual and haptic interface. Adv. Robot..

[9] Kimura Y., Yanagimachi Y. (1995). Intracytoplasmic sperm injection in the mouse. Biol. Reprod..

[10] Kim D., Kim B., Yun S., Kwon S. Cellular force measurement for force reflected biomanipulation. Proceedings of the IEEE International Conference on Robotics and Automation.

[11] Desai J., Pillarisetti A., Brooks A. (2007). Engineering Approaches to Biomanipulation. Annu. Rev. Biomed. Eng..

[12] Lu Z., Chen P., Luo H., Nam J., Ge R., Lin W. (2009). Models of maximum stress and strain of zebrafish embryos under indentation. J. Biomech..

[13] Asgari M., Abdi H., Lim C., Nahavadi S. Formulation and simulation of a 3D mechanical model of embryos for microinjection. Proceedings of the 2013 IEEE International Conference on Systems, Man, and Cybernetics, SMC.

[14] Lu Z., Chen P., Nam J., Ge R., Lin W. (2007). A micromanipulation system for automatic batch microinjection. J. Micromech. Microeng..

[15] Pillarisetti A., Member S., Pekarev M., Brooks A., Desai J., Member A. (2007). Evaluating the Effect of Force Feedback in Cell Injection. IEEE Trans. Autom. Sci. Eng..

[16] Thurgood P., Baratchi S., Szydzik C., Zhu J.Y., Nahavandi S., Mitchell A., Khoshmanesh K. (2018). A self-sufficient micro-droplet generation system using highly porous elastomeric sponges: A versatile tool for conducting cellular assays. Sens. Actuators B Chem..

[17] Xie Y., Sun D., Liu C., Tse H.Y., Cheng S. (2010). A Force Control Approach to a Robot-assisted Cell Microinjection System. Int. J. Robot. Res..

[18] Kim D., Yun S., Kim B. Mechanical force response of single living cells using a microrobotic system. Proceedings of the International Conference on Robotics and Automation.

[19] Liu X., Kim K., Zhang Y., Sun Y. (2009). Nanonewton Force Sensing and Control in Microrobotic Cell Manipulation. Int. J. Robot. Res..

[20] Chen P., Zhou S., Lu Z., Nam J., Luo H., Ge R., Ong C., Lin W. (2015). Speed optimization in automated microinjection of zebrafish embryos. Int. J. Control Autom. Syst..

[21] Sadak F., Saadat M., Hajiyavand A., Nomicos G. Vibrational Analysis During Cell Injection in ICSI Operation. Proceedings of the 2018 International Conference on Manipulation, Automation and Robotics at Small Scales (MARSS).

[22] Zhou S., Chen P., Lu Z., Hoo N., Luo H., Ge R., Ong C., Lin W. Speed Optimization for Micropipette Motion during Zebrafish Embryo Microinjection. Proceedings of the 11th International Conference on Control Automation Robotics & Vision.

[23] Zhao Y., Sun H., Sha X., Gu L., Zhan Z., Li W. (2019). A Review of Automated Microinjection of Zebrafish Embryos. Micromachines.

[24] Kim D., Hwang C., Sun Y., Lee S., Kim B. (2006). Mechanical analysis of chorion softening in prehatching stages of zebrafish embryos. IEEE Trans. NanoBiosci..

[25] Westerfield M. (1995). The Zebrafish Book: A Guide for the Laboratory Use Of Zebrafish.

[26] Hajiyavand A., Saadat M., Bedi A. Polar body detection for ICSI cell manipulation. Proceedings of the International Conference IEEE Manipulation, Automation and Robotics at Small Scales (MARSS).

[27] Mozafar S., Hajiyavand A., Singh Bedi A. (2018). Oocyte Positional Recognition for Automatic Manipulation in ICSI. Micromachines.

[28] Sun Y., Wan K., Roberts K., Bischof J., Nelson B. (2003). Mechanical property characterization of mouse zona pellucida. IEEE Trans. Nanobiosci..

[29] Abena A., Malacaris D., Miraglis A., Marulo F. Numerical simulation of a ballistic impact on the aeronautical structure in composite material. Proceedings of the Italian Association of Aeronautics and Astronautics XXII Conference.

[30] Abena A., Soo S., Essa K. (2015). A Finite Element Simulation for Orthogonal Cutting of UD-CFRP Incorporating a Novel Fibre-matrix Interface Model. Procedia CIRP.

[31] Abena A., Essa K. (2017). Modelling the orthogonal cutting of UD-CFRP composites: Development of a novel cohesive zone model. Compos. Struct..

[32] Kim D., Sun Y., Yun S., Kim B., Hwang C., Lee S., Nelson B. Mechanical property characterization of the zebrafish embryo chorion. Proceedings of the 26th Annual International Conference of the IEEE IIn Engineering in Medicine and Biology Society, IEMBS’04.

